# Spotted Lanternfly (Hemiptera: Fulgoridae) Nymphal Dispersion Patterns and Their Influence on Field Experiments

**DOI:** 10.1093/ee/nvab104

**Published:** 2021-09-23

**Authors:** D D Calvin, J Keller, J Rost, B Walsh, D Biddinger, K Hoover, B Treichler, A Johnson, R T Roush

**Affiliations:** 1 Office of the Dean, The Pennsylvania State University, University Park, PA 16802, USA; 2 Department of Entomology, The Pennsylvania State University, 437 Ag Administration Building, University Park, PA 16802, USA; 3 Department of Horticulture, Penn State Berks Campus, Tulpehocken Road, P.O. Box 7009, Reading, PA 19610, USA; 4 Penn State Extension Berks County Office, 1238 County Welfare Road # 110, Leesport, PA 19533, USA; 5 Fruit Research and Extension Center, P.O. Box 330, Biglerville, PA 17307-0330, USA; 6 U.S. Army Corp of Engineers, Blue Marsh Lake, 1268 Palisades Drive, Leesport, PA 19533, USA

**Keywords:** invasive species, aggregation, experimental design, *Lycorma delicatula*, spotted lanternfly

## Abstract

The spotted lanternfly, *Lycorma delicatula* (Hemiptera: Fulgoridae) (White, 1845), is an invasive pest in the Mid-Atlantic region of the United States. Understanding this pest’s dispersion patterns is fundamental for development of management and surveillance programs. To address this knowledge gap, we quantified spotted lanternfly nymph dispersion patterns by instar for rural and urban/suburban habitats, and we compared the number of sample units required for sticky traps and in situ visual counts to estimate population densities at several precisions. In addition, we assessed the ability of two experimental designs (completely random and randomized complete block) to detect management practices’ impacts in the field. All instars typically followed an aggregated dispersion pattern. Sample size and time requirements for checking and replacing sticky traps and for conducting in situ counts were similar, but in situ counts do not require purchasing traps, installation time, or delays before treatment, and do not remove insects. Although the cost for using in situ counts is likely less than for sticky traps, early instar spotted lanternfly nymph populations are harder to visually detect than later instars because of their small size, which may negate any cost advantage when treatments are applied early. In general, using a randomized complete block design resulted in higher statistical power than a completely random design, allowing detection of proportional population reductions of 10–20% less with equal replication. Studies aiming to evaluate treatments that reduce spotted lanternfly numbers by less than 60% will require researchers to evaluate the feasibility of using the required large sample sizes.

Efficient and effective management of invasive insects to slow their spread, protect valuable commodities, and prevent nuisance problems for homeowners requires evaluating the effectiveness of management tools, including insecticides (Brockerhoff et al. 2010). Small-scale trials under tightly controlled experimental conditions are useful to determine the efficacy of available insecticides and other management tactics (e.g., Sabbatini Peverieri et al. 2018). Large-scale field trials, however, offer a more realistic picture of the levels of control that can be achieved should candidate management practices be applied for landscape-level control (Bruck et al. 2011). Larger-scale field trials may be hampered by substantially increased heterogeneity in experimental units when compared against small-scale studies (Bartlett 1936). Additionally, complete counts of all individual insects in large field trials are impractical, requiring researchers to use other sampling techniques, such as trapping or limited search protocols, which can add further variance and uncertainty to the collected data. Finally, confinement of insects to treated areas is unrealistic in large-scale field trials, which allows individuals to leave or enter experimental plots over time, potentially obscuring the impact of treatments. With sufficient pilot study data, experimenters can design large-scale field trials with enough statistical power to detect relevant differences in pest abundance despite these hindrances.

The spotted lanternfly, *Lycorma delicatula* (Hemiptera: Fulgoridae) (White 1845), is a new invasive pest in the Mid-Atlantic region of the United States, and its rapid spread and costly negative impacts encourage pest managers to consider widespread landscape-level control (Leach et al. 2019, Lee et al. 2019). This planthopper is a voracious phloem-feeder that occupies a variety of habitats including forests and croplands. It attacks timber species and valuable agricultural commodities. Currently, grapes have been the hardest hit commodity in the United States, with death of grapevines and reduced productivity (Harper et al. 2019). The spotted lanternfly is also a major nuisance pest for homeowners because it excretes large volumes of honeydew as it feeds, which frequently promotes the growth of sooty mold (Dara et al. 2015). Honeydew and sooty mold cover plants, equipment, vehicles, porches, toys, etc., located under its host plants. Once the sooty mold develops on a substrate, it is difficult to remove.

Native to parts of eastern Asia, spotted lanternfly was first detected in Berks County, PA in September of 2014 (Barringer et al. 2015, Dara et al. 2015). Since initial detection, the pest has spread and established populations in 34 Pennsylvania, 19 New Jersey, 6 New York, 1 Connecticut, 1 Ohio, 2 Delaware, 5 Virginia, 2 West Virginia, and 4 Maryland counties as of May 2021 (NYSIPM 2021). Individual spotted lanternflies not suspected to originate from a local established population have been found at numerous other locations outside quarantined areas. When the first individual or population is reported in an area, a population may have been present and established for several years at densities that are difficult to detect. Thus, it is possible these initial sightings will lead to the discovery of new populations, expanding the pest’s range in North America.

Dispersion pattern is a fundamental attribute of biological populations and is important in the development of management programs. It is defined as the spatial arrangement of an organism’s population between habitat units within a larger habitat area at any instant in time (Brown and Orians 1970). Dispersion is typically defined as either being random, uniform, or aggregated (Lloyd 1967). A random dispersion pattern occurs when the probability of finding an individual organism is equal regardless of the presence of other individuals of the same species or life stage. A uniform dispersion pattern occurs when the presence of an individual in a habitat unit decreases the chances of additional individuals occurring in the same habitat unit. An aggregated dispersion pattern occurs when the presence of an individual increases the likelihood that additional individuals will occur in the habitat unit. Iwao (1968) outlines how dispersion patterns can be described using mathematical distributions and how the necessary sample size to estimate abundance with a given precision can then be calculated. Here, we quantify the dispersion pattern of the four spotted lanternfly instars and report sample size requirements to measure population mean densities at several levels of precision in both rural and urban/suburban settings.

In addition to quantifying spotted lanternfly dispersion, we measured several other attributes of spotted lanternfly distribution and biology relevant to designing large-scale management trials in the mixed habitat of its invaded range in the United States. These include: 1) the seasonal pattern in spotted lanternfly abundance, 2) the correlation in trap catch over time, and 3) the efficiency of two sampling techniques: sticky band traps and in situ timed visual searches. Based on this information, we calculated the sample size required to distinguish treatments with various effect sizes from an untreated control with 80% statistical power on several dates throughout the growing season. This calculation was carried out for experiments using either sticky bands or in situ visual counts to quantify spotted lanternfly abundance. We conducted a simulation study to investigate the relative power of two experimental designs: the completely random design where all plots are randomly allocated to be treated or left as a control, and the randomized complete block design with blocks determined by pretreatment spotted lanternfly counts. Together, the results presented here provide new insight into the biology of one of the most threatening emerging invasive insects in the United States and present the necessary groundwork to guide future management trials. The methodology applied here may also be adapted to guide experiments in diverse insect pest systems.

## Materials and Methods

### Phenology of Spotted Lanternfly Nymphs

We designed our sampling plans to span the development of spotted lanternfly through its four nymphal instars. In southeastern Pennsylvania, spotted lanternflies typically hatch in May and early June, with peak hatch in mid-May (Liu 2019), though weather variations may shift the timing of hatch. Prior investigations of spotted lanternfly phenology in this region recorded first instar nymphs present from hatch through early July, second instar nymphs present from early June through July, third instar nymphs present from late June through late July, and fourth instar nymphs present through July and into August (Liu 2019, Leach and Leach 2020).

### Description and Characteristics of Study Areas

**Fig. 1.**
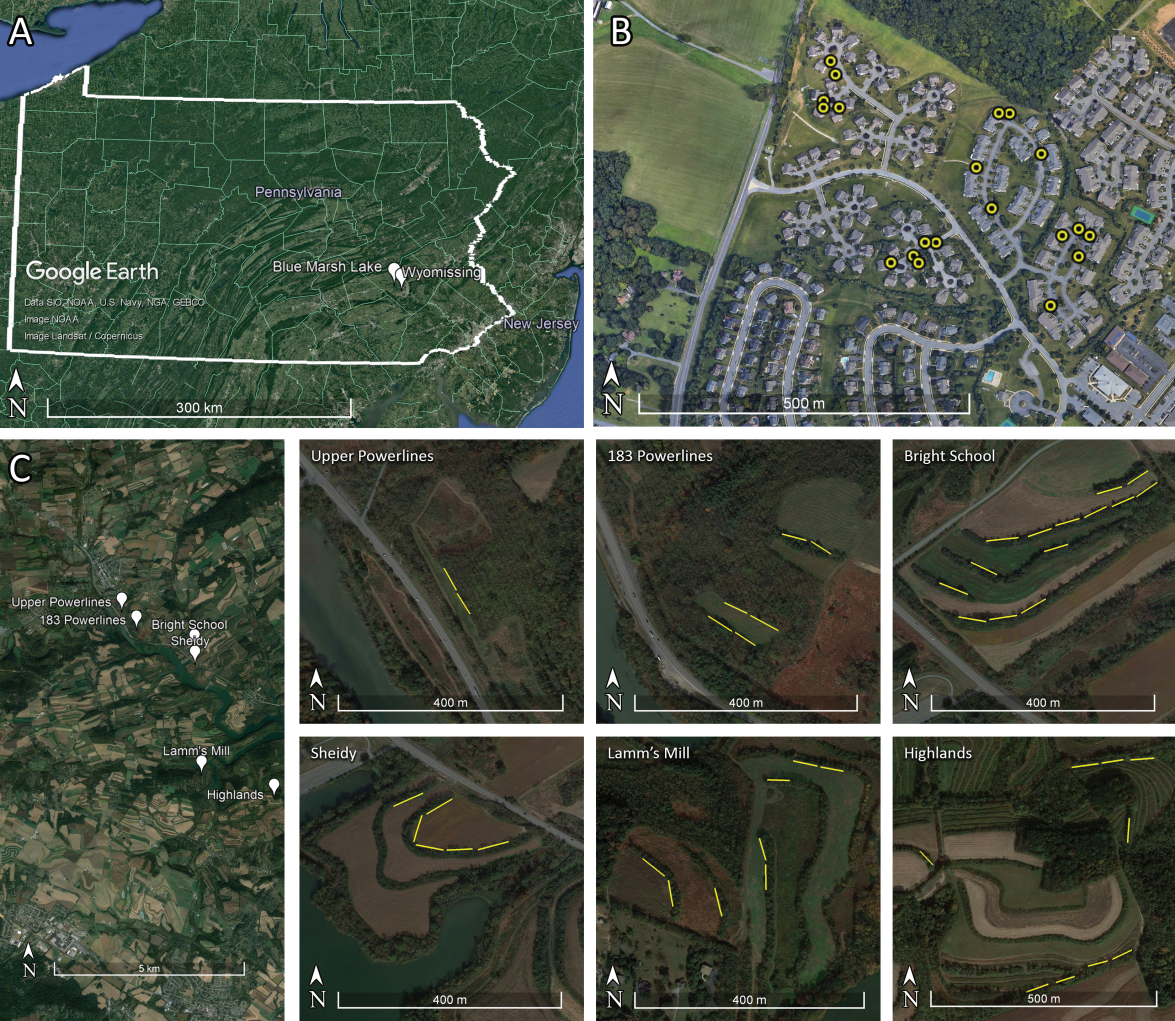
Locations of spotted lanternfly observations. Panel A shows the locations of Blue Marsh Lake and the Wyomissing housing development where we trapped and observed spotted lanternfly. Panel B shows the locations of all trapped trees within the Wyomissing housing development. Panel C shows the locations of the six sites within Blue Marsh Lake and the locations of all plots (line segments, each of which had two trapped trees) at these sites.

Nymph population dispersion studies were conducted in Berks County at a housing association in Wyomissing, PA in 2019 and 2020 and at the Blue Marsh Lake Recreation Area (‘Blue Marsh Lake’) in Berks County, PA in 2020 (Fig. 1). At Blue Marsh Lake, sampling was carried out as part of an insecticide efficacy trial. At the Wyomissing housing association, sampling was carried out as part of a study of spotted lanternfly phenology. These two sites were within the county where spotted lanternfly was first discovered in the United States.

Blue Marsh Lake Recreation Area is a typical southeast Pennsylvania rural habitat characterized by fragmented woodlots and farm fields broken by windbreaks. In contrast, our other study site at the housing association provided a typical landscaped suburban/urban habitat. Urban/suburban habitats include highly landscaped neighborhoods, parks, businesses, vacant lots, and roadways interspersed with expanses of forested woodlots and/or unmaintained natural buffer strips often comprised of fence rows remaining from previous agricultural land use prior to housing development use. A common source of spotted lanternfly in both habitat types is its highly preferred host, *Ailanthus altissima* (Mill.) Swingle (Sapindales: Simaroubaceae). In more highly landscaped urban/suburban settings, *Acer rubrum* L. (Sapindales: Sapindaceae) and *Acer saccharinum* L. (Spindales: Sapindaceae) are common preferred hosts for all spotted lanternfly life stages (Barringer and Ciafré 2020).

The suburban/urban plot areas were established to track the seasonal development of spotted lanternfly populations in a housing association with *A. rubrum* of the same age planted as street trees. The rural plots were selected within Blue Marsh Lake in soil conservation strips established in the 1970s and allowed to grow without management, often containing high densities of invasive plant species commonly fed upon by spotted lanternfly, such as *A. altissima* and *Celastrus orbiculatus* Thunb. (Celastrales: Celastraceae) as well as common native hosts such as *Juglans nigra* L. (Fagales: Juglandaceae) and *Acer* spp. (Barringer and Ciafré 2020), hereafter referred to windbreaks. The open fields either had an agricultural crop, or, if unmanaged, a mix of herbaceous and woody shrub growth.

### Experimental Layout and Sampling Protocol for Rural Locations (Blue Marsh Lake)

**Fig. 2.**
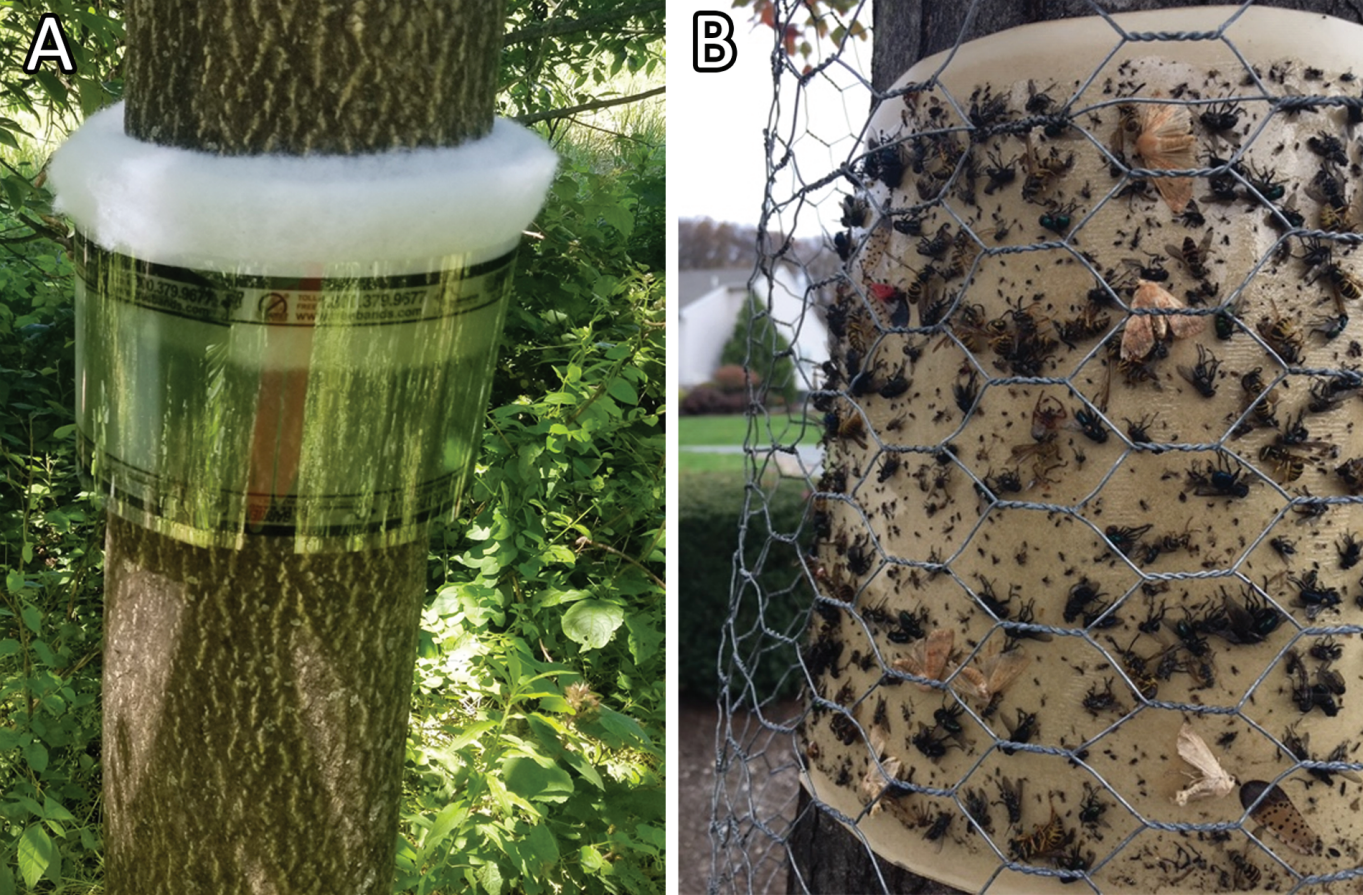
Inward-facing BugBarrier sticky band trap (A) and outward-facing sticky band trap with chicken wire to reduce bycatch (B) installed on trees.

During the 2020 season, 45 woodlot plots (46.2 m long by 15.4–30.8 m wide) with a mix of *A. altissima* and other hosts were established and sampled for spotted lanternfly nymphs at Blue Marsh Lake (Fig. 1C). These plots were used in a large-scale insecticide field trial and included nine untreated control plots. Here, we focus on nymphal dispersion patterns over plots and implications for future experiments; results of insecticide trials will be presented elsewhere. In each plot, two BugBarrier (Product #BB250 Envirometrics Systems USA, Victor, NY; Fig. 2A) sticky traps per plot (90 total) were placed on *A. altissima* trees of similar diameter at breast height (DBH). Breast height is defined as 1.4 m above ground level. In two plots, *A. altissima* trees of sufficient size for trap installation were not available, so *Prunus serotina* Ehrh. (Rosales: Rosaceae) were used instead. The average DBH of trees on which traps were installed was 17.6 ± 0.5 cm (*N* = 90). Trap surface area was estimated by adding the width of foam separating the sticky film from the tree to trees’ DBH measurement, calculating the trap circumference, and multiplying by the width of exposed sticky surface of the trap on which nymphs were collected.

The Blue Marsh Lake plots were originally laid out as part of a single experiment to evaluate insecticide efficacy, but because the plots were spread out into six unique areas throughout the recreation area, we also treated the six areas as individual sites and sticky traps (trees) within the original 45 pretreatment plots (90 traps) as sampling units to estimate the nymphal dispersion pattern between *A. altissima* trees. The number of sticky traps (sampling units) within a site varied from 4 to 28, depending on the number of plots located at each site. Across the six sites, the average number of sampling units (traps) was 15.

Table 1 provides two measures of a defined site area for the Blue Marsh Lake study in 2020. The first is a larger general geographic area that includes both the windbreaks where primary host trees exist and all the farmland and other habitat types between and around the windbreaks. The second measure captures the area of only the windbreaks within which the plots (and traps) were positioned. Google maps (https://www.google.com/maps) was used to estimate the area of each site. The trap density per hectare was calculated by dividing the total number of traps in the site by the estimated site area.

**Table 1.** Field site details for study at Blue Marsh Lake Recreation Area, Leesport, PA in 2020

Location name	GPS location	General area		Sample units	Units (traps) per hectare	Windbreak hectares	Units (traps) per windbreak hectare
		Area (m^2^)	Hectares				
Bright School	40.415636 N −76.086004 W	129,572.1	12.96	28	2.20	3.50	8.00
Sheidy Boat Launch	40.084375 N −76.084375 W	37,833.1	3.78	12	3.17	2.20	5.45
Powerline	40.418854 N −76.101825 W	49,957.9	4.96	12	2.42	1.00	4.96
Upper Powerline	40.424817 N −76.081655 W	10,308.9	1.03	4	3.88	0.41	9.76
Lamm’s Mill	40.385035 N −76.081655 W	177,173.4	17.70	16	0.90	0.99	16.16
Highlands	40.380540 N −76.057152 W	226,997.5	22.70	18	0.80	2.70	6.67
Total or (Ave)		631,842.9	63.13	90	(2.22)	10.8	(6.87)

Except for two traps placed on *P. serotina* trees, all traps were placed on *A. altissima* trees within windbreak areas.

Sticky traps were first placed on 8 June 2020. The number of nymphs captured per trap (tree) was then assessed on 11, 14, 23, and 26 June and 4, 7, 10, 18, 21, 24, 27, and 30 July 2020. On each observation date, the adhesive film of each trap was removed from the tree and the number of nymphs of each instar was counted and recorded. The old adhesive films were then replaced with new ones. All traps were left in place for 3 d prior to collection and counting; there were some days during the sampling period on which traps were not in place due to treatment applications. Final counts were adjusted to the number of nymphs per 1,000 cm^2^ of trap area.

In addition to monitoring spotted lanternfly abundance using sticky bands, we also carried out timed in situ visual counts, with four observers each counting all individuals they could see from a single point within the plot during a 5-min observation period. This was a slight variation of a method developed by one of us (R.T.R.) in 2019, in a successful test of a biological insecticide (Clifton et al. 2020). These counts occurred after the nymphs had grown large enough (at least second instar) that we could see them reliably, on 2, 9, 16, and 23 July. Timed in situ visual counts occurred on different days than sticky band trap observations due to staffing and time constraints.

### Sampling Protocol for Suburban/Urban Housing Association (Wyomissing, PA)

At the Wyomissing location, the site was an area of approximately 18 ha; thus, the site was defined as the area covered by the housing association. Within the housing association, we installed outward-facing, 20.3-cm wide brown sticky traps that spanned the circumference of the tree on 19 *A. rubrum* and one *Quercus palustris* Münchh. (Fagales: Fagaceae) across the housing development (Fig. 2B). The trees ranged in height from 5.5 to 16.8 m and their DBH ranged from 7.6 to 40.6 cm. Sampling units were defined as individual sticky traps on a tree or 1,000 cm^2^ of a sticky trap area. Each sticky trap was surrounded by chicken wire to prevent the accidental capture of nontarget vertebrates. The difference in sticky band traps between suburban and rural sampling locations is incidental, as these two sampling plans were not initially conceived as two portions of the same study. We note that both types of sticky band perform comparably in the field, especially for early instar nymphs (Francese et al. 2020).

The number of captured nymphs of each instar was documented once weekly beginning on 25 May 2019 and 1 June 2020. In 2019, samples were collected over 24 wk, ending on 12 November 2019. In 2020, samples were collected over 25 wk ending on 17 November 2020.

### Quantification of Nymphal Dispersion Patterns and Sample Number Curves

Because population density data do not always follow a random or normal distribution, particularly when sample size is low, we applied the methodology from Iwao and Kuno (1968). Using this approach, the sample size necessary to estimate spotted lanternfly abundance with a given precision was calculated for all instars for Blue Marsh Lake and for Wyomissing. This approach can be applied broadly without the restriction of an underlying mathematical distribution (Iwao 1968, Elliot 1977).

Calvin et al. (1986) described the process of estimating dispersion patterns and generating sample number curves. The first step is to calculate mean crowding of the population as described by Lloyd (1967). This approach uses the population sample mean density per habitat unit and the ratio between the calculated sample population mean density and its variance across habitat units. For this study, a habitat unit was defined as a sticky trap (tree) or 1,000 cm^2^ of sticky trap area. While traps are not true ecological habitat units, they provide a method for making a relative estimate of the population mean density that can be used for pest management or surveillance purposes. Campbell et al. (2002) used traps to determine the spatial arrangement of several stored grain insect species over time. Yellow sticky traps were used by Midgarden et al. (1993) to estimate the dispersion pattern of western corn rootworm (*Diabrotica virgifera virgifera* LeConte (Coleoptera: Chrysomelidae)) adults and develop sample number requirements.

The slope parameter from a linear regression between mean density and mean crowding was used as a measure of aggregation across traps within a site. Using this approach, a slope parameter of one indicates that the population is generally randomly distributed and follows a Poisson distribution. A slope that is significantly less than 1.0 indicates that the population density is more uniform across habitat units and follows a positive binomial mathematical distribution. A slope that is greater than 1.0 indicates the population is aggregated across habitat units and follows a negative binomial mathematical distribution. As stated earlier, Iwao and Kuno (1968) showed that the relationship held under several mathematical distributions, and it is commonly used to describe insect dispersion patterns. The mean density, variance, and mean crowding were calculated using Excel 365 (Microsoft, Redmond, WA) for each treatment and date of sampling in 2020 at Blue Marsh Lake and Wyomissing in 2019 and 2020. We also calculated these values for each instar separately and for all instars combined.

The index of dispersion (*I* = *s*^2^/*x*) described by Morisita (1959) was used as an indicator of the spotted lanternfly instars’ dispersion pattern between traps within a site. Elliott (1977) described a method to test departure from unity, which is consistent with a random distribution. The test consists of calculating a χ ^2^ value for each site using the equation:


$$\begin{array}{l} \chi^{2}=I(n\;-\;1) \end{array}$$ (1)

where *I* = index of dispersion and *n* = number of sampling units. The resulting χ ^2^ values were tested against the upper and lower confidence limits for a χ ^2^ value with *n* − 1 degrees of freedom.

Sample number (*q*) curves for various population densities were calculated for both locations at three levels of precision using the formula:


$$\begin{array}{l} q=t^{2}/d^{2}\{[(\alpha +1)/x]+(\beta \;-\;1)\} \end{array}$$ (2)

where *t* = Student’s *t*-test (df = 1.96; *P* = 0.05); *d* = desired level of precision; α = y-axis intercept obtained from the linear regression model, and β = slope obtained from the regression model (Iwao and Kuno 1968). Because the sampling method was developed for extensive use, a maximum precision level of 0.25 was used (Southwood 1971). A precision level of 0.30 was included to illustrate the effect of lowering precision on the number of samples necessary to obtain an adequate estimate of the population density.

A similar analysis was conducted to compare the degree of aggregation before and after application of insecticide treatments. To assess dispersion in all plots prior to treatment, we fit a regression of mean density against mean crowding using data from across the seven treatments on the two observation dates prior to the application of treatments. To measure dispersion in all observations of untreated plots, we added data from untreated control plots through the rest of the season, which included 11 more observation dates, and fit another regression using these data. To measure dispersion in all plots, regardless of whether treatment was applied or not, we fit a regression for mean density against mean crowding for all observations across the entire study. We then compared fitted regression parameters to assess whether dispersion pattern varied across these three data sets.

### Examining Seasonal Correlation in Spotted Lanternfly Catch on Sticky Bands

To measure consistency in the relative ranking of traps’ catch over time, we calculated Spearman’s rank correlation coefficient comparing traps’ catch on each observation date from 14 June 2020 forward against their catch on 11 June 2020 for all traps in control plots in the Blue Marsh Lake experiment. We also calculated Spearman’s rank correlation coefficients comparing each observation date’s counts against the prior observation date’s counts. The number of days between observations varied from 3 to 8 d, but all traps were left in place for 3 d prior to observation.

### Estimating the Number of Sample Units (Replications) Needed to Detect a Treatment Effect (Difference Between Treatments) for Sticky Traps and In Situ Counts

While the Iwao and Kuno (1968) approach is designed to calculate the number of sample units needed to estimate the true population density at a given level of accuracy and precision without assuming a mathematical distribution, it is not designed to estimate the number of samples (replications) required to measure differences between two groups. To estimate the sample size required to detect differences in mean trap catch between untreated control plots and plots treated with a hypothetical insecticide known to reduce pest numbers by a given percentage, we began by taking the natural logarithm of the count data from control plots, adding 1 to all counts to prevent undefined values for traps with 0 individuals. Taking the natural logarithm allowed us to perform power analysis on a scale where negative counts could not occur, as they cannot occur in the field collected data.

We confirmed the normality of log-transformed counts by visual inspection of quantile–quantile plots for each date’s observations. We then calculated the shift in the mean on the log scale that resulted in each desired proportionate decrease in mean count on the untransformed count scale as follows:


$$\begin{array}{l} j=\;log(1-r) \end{array}$$ (3)

where *j* is the difference between means on the log scale and *r* is the desired proportional decrease in mean counts. Then, for each date, we estimated the sample size required to achieve a power of 80% for a one-sided *t*-test comparing the means of control and treated plots using the *pwr.t.test* function from the *pwr* package in R (Champely 2020, R Development Core Team 2020). We calculated sample size requirements for proportional changes of 20, 30, 40, 50, 60, 70, 80, and 90% for 4, 10, 18, and 24 July 2020. We repeated this process using the sum of four observers’ 5-min timed visual counts in each plot as an alternative method for quantifying spotted lanternfly abundance.

### Examining the Effect of Blocking by Initial Spotted Lanternfly Abundance on Experiments’ Power Using a Simulation Approach and Including the Measured Decline in Population Mean Density Between Pretreatment and Posttreatment Counts

To determine whether an experimental design with plots blocked by spotted lanternfly abundance prior to treatment would have greater power than a completely random design, we conducted a series of simulations. To begin, we based these simulations on data collected using BugBarrier sticky band traps in the insecticide trial experiment conducted at Blue Marsh Lake. For each series of simulations, we selected the number of replicates (*n*) to be simulated and the proportional reduction in spotted lanternfly abundance caused by the treatment of interest (*r*). We also selected the date on which the assessment of the treatment’s effect occurred. Initial spotted lanternfly abundance (*N*_init_) was simulated by fitting a negative binomial distribution (linear parameterization as in Hardin and Hilbe 2007) to the total spotted lanternfly catch counts from the observation date prior to the date of assessment and drawing 2*n* random numbers from the fitted distribution.

Each of these simulated plots was then assigned to either be treated or left as an untreated control following two methods. In the completely random design, equal numbers of plots were assigned to each group at random. In the blocked design, simulated plots were sorted from highest to lowest initial spotted lanternfly abundance, and then plots in each sequential group of two were randomly allocated to the control or the treatment.

To project forward and give each simulated plot a count on the preselected date of assessment, we used the *glmmTMB* function from the *glmmTMB* package (Brooks et al. 2017) to fit a negative binomial regression, regressing field collected data describing spotted lanternfly count on the assessment date in untreated control plots against spotted lanternfly count on the prior observation date in those plots. We then assigned each simulated plot a posttreatment spotted lanternfly count based on this regression under each experimental design (completely random and blocked). For plots assigned to the untreated control, we drew a random number from the negative binomial distribution with mean μ = β*N*_init_ and variance = μ * (1 + φ), where β is the fitted slope from the regression of count on assessment against count on the prior date, and φ = μ/*k*, where *k* is the fitted overdispersion parameter from the regression. For simulated plots assigned to be treated with insecticide, we reduced the mean by multiplying by (1 − *r*). Each simulated plot then had an initial count, a simulated posttreatment count under the completely random design, and a simulated posttreatment count under the blocked design.

We then fit two negative binomial regressions to see whether the simulation detected the difference we imposed between the control and treated plots. For the completely random design, we regressed posttreatment counts against treatment group, and determined significance based on a one-tailed *t*-test assessing whether the treated plots’ mean spotted lanternfly count was less than that of the control plots. For the blocked design, we added a random effect for block to the model and determined significance in the same manner. This full process was carried out 300 times for each combination of assessment date (14 and 23 June and 4, 10, and 21 July 2020), proportional reduction due to insecticide application (0.35, 0.4, 0.45, 0.5, 0.55, 0.6, 0.65, 0.7, 0.75, 0.8, 0.85, and 0.9) and number of replicates (3 through 16, 25, and 50) of interest. We estimated power for each set of parameters by dividing the number of cases where a significant difference was found by the number of simulations (300). For a step-by-step walkthrough of the simulation procedure with plots showing the steps, see the Supp Material (online only).

We repeated this process using data from the in situ visual counts carried out at Blue Marsh Lake. Assessment dates for in situ visual count data were 9, 16, and 23 July 2020. To aid regression model convergence, we scaled the values for spotted lanternfly count on the date prior to assessment before fitting the regression of spotted lanternfly counts on the assessment date in untreated control plots against spotted lanternfly counts on the prior observation date.

### Comparison of Sampling Efficiency Between Sticky Trap and In Situ Evaluation Methods

The time required to monitor sticky traps and count instar numbers was recorded in 2020 at Wyomissing. The cost to monitor a trap (tree) was calculated for a personnel cost of $12.50, $15.00, and $20.00 per hour. We estimated the labor cost to service traps based on records of time spent checking traps at both the suburban trees in Wyomissing and the rural trees at Blue Marsh Lake. We also estimated the labor cost for conducting timed in situ counts. Using this information, an estimate of relative efficiency between methods was calculated. We did not include the time spent traveling between plots because this was the same for both methods.

## Results

### Blue Marsh Lake Rural and Wyomissing Housing Association Urban/Suburban Site Measurements

At Blue Marsh Lake, sites (combined windbreak areas and other habitat) varied in size from 1.03 to 22.7 ha, averaging 10.5 ha. This area was partitioned out of an area of mixed forested woodlot, windbreaks, and open nonforested areas (Fig. 1). The windbreak areas (excluding other habitat between plots) ranged in size from 0.41 to 3.5 ha, averaging 1.8 ha. Based on these measurements for the sites, the density of traps was equivalent to 0.8–3.88 traps per hectare (mean = 2.22 traps per hectare) for the larger site scale and 4.96–16.16 traps per hectare (mean = 6.87 traps per hectare) for the windbreak area only sites. The total area of the Wyomissing site was approximately 18 ha.

### Dispersion Patterns of Spotted Lanternfly Nymph Stages Using Sticky Traps

#### Urban/Suburban A. rubrum Trees

At the Wyomissing housing association, nymphs had an aggregated dispersion pattern across all four instars. Table 2 provides the intercept (SE) and slope (SE), the *R*^2^ value, *F*-value, and Pr > *F* for the linear relationship between mean density and mean crowding for each spotted lanternfly nymph stage captured on individual *A. rubrum* trees (traps) and per 1,000 cm^2^ of trap area for an urban/suburban housing association in Wyomissing. Slope values in parentheses are for linear regressions that force the relationship through the origin (Waters et al. 2014).

**Table 2.** Linear regression results for the relationship between mean density and mean crowding per tree and per 1,000 cm^2^ of trap area for spotted lanternfly instars for urban *A. rubrum* at Wyomissing, PA in 2019 and 2020

Instar	Slope (± SE)	Intercept (± SE)	R^2^ value	F-value	Pr > F
Per tree (trap)					
First	1.39 ± 0.06	71.40 ± 53.38	0.981	470.0	<0.0001
	(1.43 ± 0.06)		0.984	626.4	<0.0001
Second	1.56 ± 0.15	15.24 ± 7.07	0.872	116.9	<0.0001
	(1.73 ± 0.13)		0.906	173.5	<0.0001
Third	2.51 ± 0.19	−0.23 ± 1.59	0.924	184.2	<0.0001
	(2.49 ± 0.13)		0.957	353.4	<0.0001
Fourth	2.56 ± 0..26	0.41 ± 0.93	0.857	96.6	<0.0001
	(2.64 ± 0.18)		0.926	212.6	<0.0001
Combined	1.41 ± 0.03	21.81 ± 13.81	0.991	2745.4	<0.0001
	(1.43 ± 0.03)		0.991	3039.7	<0.0001
Per 1,000 cm^2^ of trap area					
First	1.42 ± 0.07	44.2 ± 35.6	0.979	413.4	<0.0001
	(1.46 ± 0.06)		0.983	566.1	<0.0001
Second	1.58 ± 0.14	8.66 ± 4.21	0.883	129.8	<0.0001
	(1.75 ± 0.13)		0.914	192.0	<0.0001
Third	2.53 ± 0.17	−0.77 ± 0.87	0.939	230.5	<0.0001
	(2.43 ± 0.12)		0.960	385.2	<0.0001
Fourth	2.99 ± 0.32	−0.54 ± 0.70	0.842	86.0	<0.0001
	(2.82 ± 0.22)		0.899	151.0	<0.0001
Combined	1.44 ± 0.03	13.20 ± 9.26	0.989	2388.1	<0.0001
	(1.46 ± 0.03)		0.990	2689.2	<0.0001

Values in parentheses are for regressions without a fitted y-intercept, where the regression line is forced through the origin (0, 0).

Slope and intercept values, *R*^2^ values, and Pr > *F* were similar when expressed as number per 1,000 cm^2^ of trap area. When the relationships were forced through zero, the slope values were similar to those obtained without forcing it through zero, but *R*^2^ values were slightly higher due to one additional degree of freedom in the fit since only one parameter was estimated.

#### Rural *A. altissima*

In the rural setting a Blue Marsh Lake, nymphs had an aggregated dispersion pattern across all four instars, and they had broadly similar levels of aggregation to those caught on bands in the urban/suburban sampling in Wyomissing. Table 3 provides the intercept (SE) and slope (SE), the *R*^2^ value, *F*-value, and Pr > *F* for the linear relationship between mean density and mean crowding for each spotted lanternfly nymph stage captured on individual *A. altissima* trees (traps) and per 1,000 cm^2^ of trap area for rural windbreaks at Blue Marsh Lake. In parentheses are the slope, SE of the slope, *R*^2^, *F*-value, and Pr > *F*-value for regressions forced through the origin, that is, with no fitted y-intercept.

**Table 3.** Linear regression results for the relationship between mean density and mean crowding per tree and per 1,000 cm^2^ of trap area for spotted lanternfly nymph instars, for *A. altissima* in windbreak areas at Blue Marsh Lake in 2020

Instar	Slope (± SE)	Intercept (± SE)	R^2^ value	F-value	Pr > F
Per tree (trap)					
First	2.11 ± 0.14	5.65 ± 5.20	0.813	218.6	<0.0001
	(2.18 ± 0.13)		0.852	294.2	<0.0001
Second	2.46 ± 0.21	6.27 ± 3.85	0.673	132.6	<0.0001
	(2.67 ± 0.17)		0.790	245.2	<0.0001
Third	2.42 ± 0.21	1.18 ± 1.13	0.723	136.4	<0.0001
	(2.57 ± 0.15)		0.857	312.1	<0.0001
Fourth	1.39 ± 0.17	7.58 ± 2.83	0.604	65.0	<0.0001
	(1.68 ± 0.15)		0.753	131.8	<0.0001
Combined	1.98 ± 0.15	13.48 ± 6.55	0.701	171.8	<0.0001
	(2.20 ± 0.11)		0.841	392.0	<0.0001
Per 1,000 cm^2^ of trap area					
First	2.29 ± 0.16	9.32 ± 10.70	0.797	197.9	<0.0001
	(2.35 ± 0.14)		0.839	265.9	<0.0001
Second	2.42 ± 0.23	13.48 ± 7.30	0.632	110.7	<0.0001
	(2.68 ± 0.18)		0.764	211.7	<0.0001
Third	2.39 ± 0.23	4.57 ± 2.65	0.664	109.8	<0.0001
	(2.65 ± 0.17)		0.804	231.1	<0.0001
Fourth	1.43 ± 0.20	16.20 ± 5.95	0.55	53.1	<0.0001
	(1.75 ± 0.17)		0.718	110.7	<0.0001
Combined	2.08 ± 0.17	27.11 ± 13.53	0.658	141.32	<0.0001
	(2.33 ± 0.13)		0.818	334.7	<0.0001

Values in parentheses are for regressions without a fitted y-intercept, where the regression line is forced through the origin (0, 0).

For *A. rubrum* traps in the housing association, the first and second instar tended to be slightly less aggregated than the third and fourth instar. At the Blue Marsh Lake location dominated by *A. altissima*, there was less distinction between the degree of aggregation across instars, but the second and third instars had slightly higher slope values than did the first and fourth instars, suggesting a greater degree of aggregation for these life stages. Field observations of nymph clustering within host plants were consistent at both locations, noting a higher degree of aggregation in the third instar and an even greater degree of aggregation in the fourth instar, though this clustering on particular locations within a plant was not measured by our sampling techniques.

#### Dispersion Pattern in Untreated Controls Versus All Treatments Combined

Nymphs showed similar levels of aggregation in untreated plots and in plots where treatments were applied to reduce overall numbers. Table 4 provides a comparison between the dispersion pattern measured for all plots pretreatment (11 and 14 June 2020), all plots pretreatment plus untreated control plots over the season (14 sample dates—11 June 2020 to 30 July 2020), and all plots (untreated control and treated plots) over the entire season (7 treatments × 14 sample dates). Slope values in parentheses are for regression between mean density and mean crowding when the relationship was forced through zero. The slope parameters for the regression of mean density versus mean crowding were 1.79, 1.74, and 1.76 for the pretreatment plots only (11 June and 14 June 2020), all pretreated plots plus all untreated control plots on the 12 additional sample dates, and all treatment plots on all sample dates (7 treatments × 14 dates), respectively, for the regressions that were forced through zero. *R*^2^ values were 0.92, 0.92, and 0.90. Given the closeness of these slope parameters, the application of treatments did not appear to greatly alter the degree of aggregation of spotted lanternfly nymphs across the plots. Similar degrees of aggregation (slope parameter values) were seen for the same comparisons when not forced through zero, but ranged from 1.72 for all treatments and dates to 2.16 for the pretreatment plots only.

**Table 4.** Linear relationship between mean density and mean crowding for sticky traps calculated across six replications for the seven treatments determined posttreatment in the 2020 Blue Marsh Lake insecticide efficacy study

Treatment	Slope ± SE	Intercept ± SE	R^2^ value	F-value	df	Combination
Pretreatment plots (11 and 14 June)	2.16 ± 0.35	−74.22 ± 63.75	0.758	37.5	12	<0.0001
	(1.79 ± 0.14)	(0)	(0.924)	157.3	13	<0.0001
Pretreatment plus all untreated control across all dates	1.95 ± 0.20	−35.90 ± 29.95	0.817	93.9	23	<0.0001
	(1.74 ± 0.11)	(0)	(0.923)	263.2	24	<0.0001
All treatments and all dates	1.72 ± 0.08	4.33 ± 7.78	0.811	388.1	89	<0.0001
	(1.76 ± 0.06)	(0)	(0.897)	903.1	90	<0.0001

### Dispersion Index

#### Urban/Suburban Sites

Table 5 shows the frequency with which sites had each instar dispersed randomly, uniformly, or aggregated with both the number per tree and the number per 1,000 cm^2^ of trap area as sampling units. Using the number per tree as the sampling unit, in 2019 most sites showed all four instars following an aggregated pattern, but a few low-density sites were randomly dispersed. When the instars were combined, 10 of 14 sites had an aggregated dispersion pattern, with the remaining four being randomly dispersed. When the sampling unit was adjusted to the number of nymphs per 1,000 cm^2^ of trap area, a slightly higher number of sites showed the dispersion pattern to be random versus aggregated. No sites for any instar had nymphs uniformly dispersed. The results in 2020 were very similar to 2019.

**Table 5.** Number of observations (sites × date combinations) that fit a random, uniform, or aggregated dispersion pattern based on Morisita (1959) and Elliot (1977) for each instar sampled at Wyomissing, PA housing association in 2019 and 2020

Instar	Random	Uniform	Aggregated
2019			
Per trap (tree)			
First	0	0	4
Second	2	0	7
Third	1	0	6
Fourth	1	0	8
Combined	0	0	14
Per 1,000 cm^2^ of trap area			
First	0	0	4
Second	3	0	6
Third	3	0	4
Fourth	5	0	4
Combined	4	0	10
2020			
Per trap (tree)			
First	0	0	7
Second	3	0	6
Third	1	0	7
Fourth	2	0	6
Combined	1	0	13
Per 1,000 cm^2^ of trap area			
First	1	0	6
Second	3	0	6
Third	4	0	5
Fourth	4	0	4
Combined	3	0	11

#### Rural Sites

Table 6 shows the number of sites that had each instar dispersed randomly, uniformly, or aggregated using both trees and 1,000 cm^2^ of trap area as sampling units. Using trees as sampling units again showed all four instars as following a generally aggregated pattern, but lower density sites were more randomly dispersed. When the instars were combined, 71 of 74 sites had an aggregated dispersion pattern, with the remaining three being randomly dispersed. In contrast to the results we found from monitoring the suburban *A. rubrum* trees, when we used 1,000 cm^2^ of trap area as our sampling unit, a slightly lower number of sites showed the dispersion pattern to be random versus aggregated for *A. altissima*. No sites for any instar had nymphs uniformly dispersed.

**Table 6.** Number of observations (site × date combinations) that fit a random, uniform, or aggregated dispersion pattern based on Morisita (1959) and Elliot (1977) for each instar sampled at Blue Marsh Lake Recreation Area in 2020

Instar	Random	Uniform	Aggregated
Per trap (tree)			
First	19	0	32
Second	13	0	51
Third	14	0	42
Fourth	5	0	37
Combined	3	0	71
Per 1,000 cm^2^ of trap area			
First	14	0	47
Second	8	0	56
Third	10	0	46
Fourth	3	0	39
Combined	1	0	73

### Sample Number Requirements for Estimating a Mean Density for a Population

Table 7 shows the sample number requirements for mean densities ranging from 10 to 250 total nymphs (combined instars) per tree or per 1,000 cm^2^ of trap area for both the urban/suburban and rural sites for 20, 25, and 30% precision and 5% accuracy (α = 0.05). Because the nymphs were more aggregated in the rural windbreaks than the urban/suburban housing association, the number of samples required to estimate the population mean density was approximately two times greater. For example, at 20% precision, to estimate a mean density of 100 nymphs per trap, 40 traps (PTU) or 43 1,000 cm^2^ of trap area (PCMU) were required compared to 95 traps (PTR) or 105 (PCMR) 1,000 cm^2^ of trap area. This relationship was consistent across the three precision levels.

**Table 7.** Estimated no. of traps or 1,000 cm^2^ of trap required to measure a mean density of a spotted lanternfly nymph population (all instars combined) within a site at 20, 25, and 30% precision and accuracy at α = 0.05 using the Iwao and Kuno (1968) method to calculate sample size for the Blue Marsh Lake Recreation Area and Wyomissing housing association

Mean density (no. per trap or 1,000 cm^2^ of trap)	Precision level (%)											
	20				25				30			
	PTU	PCMU	PTR	PCMR	PTU	PCMU	PTR	PCMR	PTU	PCMU	PTR	PCMR
10	49	52	104	113	31	33	66	73	22	23	46	50
25	43	46	98	108	28	30	63	69	19	20	44	48
50	41	44	96	106	26	28	62	68	18	20	43	47
75	41	44	95	105	26	28	61	67	18	19	42	47
100	40	43	95	105	26	28	61	67	18	19	42	46
125	40	43	95	104	26	28	61	67	18	19	42	46
150	40	43	95	104	26	27	61	67	18	19	42	46
175	40	43	95	104	26	27	61	67	18	19	42	46
200	40	43	95	104	26	27	61	67	18	19	42	46
225	40	43	95	104	26	27	61	67	18	19	42	46
250	40	43	95	104	26	27	61	67	18	19	42	46

PTU = per trap urban/suburban; PCMU = per 1,000 cm^2^ urban/suburban; PTR = per tree rural: PCMR = per cm 1,000 cm^2^ rural.

The number of samples that would be required to estimate the mean density of the population by instar on each sample date during the Blue Marsh Lake insecticide efficacy trial is shown in Table 8. Across the 14 sample dates during the study, the average mean density in the untreated control plots ranged from 12 to 234 total nymphs (combined) per 2 traps or per research plot. In the Wyomissing study, which had a longer interval between trap installation and replacement, average density per trap ranged from a few individuals to just under 2,000 per trap.

**Table 8.** Number of sticky trap (trees) sampling units required to estimate the mean density of the population at accuracy (α = 0.05) and precision = 0.25 and 0.30

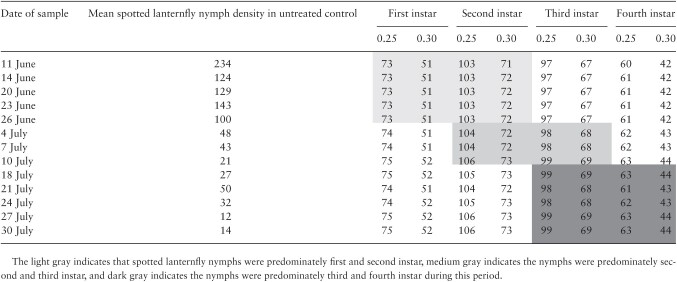

Using the mean densities from the Blue Marsh Lake Recreation Area as the 11 June 2020 density for a given instar, 73, 103, 97, and 60 traps would be needed to estimate a mean density of 234 first, second, third, or fourth instars per trap at 25% precision. The number of samples required at 30% precision would be 51, 71, 67, and 42 for the first, second, third, or fourth instars, respectively. Interestingly, measuring the lowest mean density (12 per trap on 27 July 2020) only required a few more traps at a given precision level than the number required at a mean density of 234 nymphs per trap. This suggests that within the range of mean densities observed from 11 June 2020 to 30 July 2020, the number of traps required for a given instar was relatively constant. Changes in aggregation as nymphs developed from the first to fourth instar may have a slight effect on the number of samples required, but the sample size requirement based on the total spotted lanternfly count (combining all life stages) did not vary much throughout the season. Using the relationship between mean density and mean crowding based on total nymph counts to estimate sample size (Table 7) is likely the best approach, and doing so would not have a great influence on the precision of the estimate as compared to an approach adjusting the sample size for each instar.

### Comparison Between Sticky Trap and Visual In Situ Sample Size (No. of Replications) Estimates to Detect Differences Between Treatments, Assuming a Log-Normal Distribution and Completely Random Assignment of Treatments

Estimated numbers of replications needed to detect a given percentage difference from the mean of the untreated control for a treatment effect are shown in Table 9 for sticky traps and Table 10 for in situ visual counts. Comparisons were made between similar sample dates to show how the sampling method influenced sample size (replication) requirements.

**Table 9.** Estimation of sample size of sticky traps required to reliably detect differences in spotted lanternfly abundance between untreated control plots and treated plots with varying effect size (% difference) based on four dates’ observations of the experiment at Blue Marsh Lake in 2020 using a lower one-tailed *t*-test at α = 0.05 and power = 0.80 on log-transformed counts

% Difference	4 July		10 July		18 July		24 July	
	MD (24.5)	SD (25.7)	MD (13.7)	SD (14.6)	MD (19.1)	SD (23.0)	MD (17.1)	SD (20.3)
20	294		379		471		282	
30	116		149		184		111	
40	57		73		90		54	
50	31		40		40		30	
60	18		23		29		17	
70	11		14		17		10	
80	6		8		10		6	
90	4		4		5		4	

Mean densities and standard deviation for sticky traps in the untreated control plots on 4, 10, 18, and 24 July are listed as well.

**Table 10.** Estimation of sample size of in situ visual counts required to reliably detect differences in spotted lanternfly abundance between untreated control plots and treated plots with varying effect size (% difference) based on four dates’ observations of the experiment at Blue Marsh Lake in 2020 using a lower one-tailed *t*-test at α = 0.05 and power = 0.80 on log-transformed counts

% Difference	2 July		9 July		16 July		23 July	
	MD (5.98)	SD (6.26)	MD (5.91)	SD (4.90)	MD (10.03)	SD (9.53)	MD (9.39)	SD (10.25)
20	317		277		343		228	
30	124		109		134		90	
40	61		53		66		4	
50	33		29		36		24	
60	19		17		36		14	
70	12		10		21		9	
80	7		6		13		5	
90	4		4		7		3	

Mean densities and standard deviation for in situ counts in the untreated control plots on 2, 9, 16, and 23 July are listed as well.

#### Sticky Traps

On 4 July, the number of samples required for sticky traps varied from 294 to 4 to detect a 20% and 90% difference between treatments, respectively (Table 9). On 10 July 2020, the number of samples required was slightly higher but varied similarly. The sample size requirements were higher again for the 18 July 2020 sample date. The number of replicates required to measure differences under 50% become very large. Such larger experiments may not be feasible and/or cost-effective given the spatial variation in spotted lanternfly nymph populations under field conditions. Thus, either a more efficient plot assessment approach or a better experimental design is needed that can reduce the overall experimental variation to increase the sensitivity of spotted lanternfly field studies.

#### In Situ Counts

In situ (visual) counts were conducted on similar dates (2, 9, 16, and 23 July 2020) to test whether the amount of count variation could be reduced and provide greater sensitivity in measuring treatment differences than was achieved using sticky bands. Table 10 provides a summary of sample size estimates based on mean and standard deviation values for visual counts across plots.

On 2 July, the number of samples required to measure a 20% and a 90% difference between treatments varied from 317 to 4, respectively. The 9 July 2020 and 23 July 2020 in situ count and trap sample requirements were similar to 2 July. On 16 July 2020, the number of samples needed was slightly higher. This analysis suggests that the level of sensitivity using sticky traps and in situ (visual) counts was similar.

### Trends and Correlation in Trap Catch Over Time

**Fig. 3.**
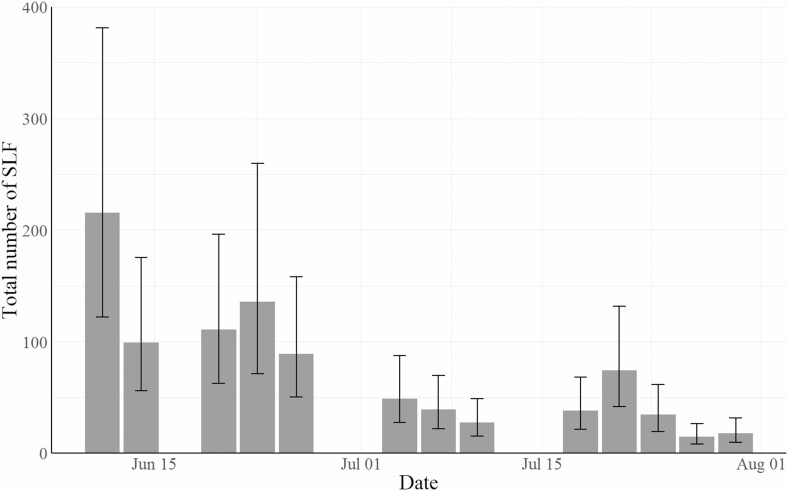
Average number of spotted lanternfly nymphs (combined instars) captured in BugBarrier sticky traps on each sample date in the untreated control plots at Blue Marsh Lake Recreation Area in 2020. Bars show estimated marginal means for a negative binomial regression of plots’ total spotted lanternfly count against date as a factor, and error bars show 95% confidence intervals for the mean.

The average number of nymphs per trap in the untreated controls declined over the sample dates during the Blue Marsh Lake experiment (Fig. 3). Although not shown in this paper, a similar decline was seen at the Wyomissing site in both study years. It is not clear if this decline is due to natural mortality, movement away from the plot areas, removal by the traps, or a combination of these three factors. This finding does suggest, however, that this decline over time must be considered when designing and executing field research.

**Fig. 4.**
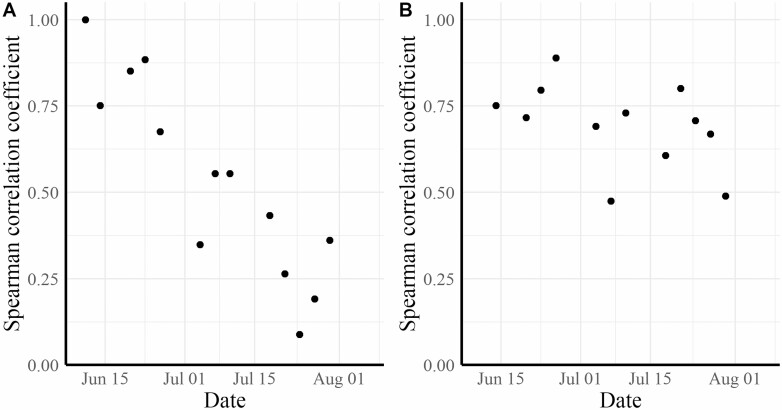
Spearman correlation coefficients between the number of nymphs per trap on June 11 (first sample date of experiment) and subsequent number of nymphs on the same trap only on each of the following sample dates (A) and Spearman correlation coefficients between the number of nymphs captured per trap and the number captured on the same trap on the previous observation, 3–8 d earlier (B).

The Spearman correlation coefficient between trap catch on a given date and trap catch on the first date of observation (11 June 2020) generally declined as the time span between counts increased (Fig. 4A). This suggests that ranking of plot spotted lanternfly densities to establish treatment blocks or pairs for assignment of treatments could be a valuable way to increase the study sensitivity if treatment effects will be measured shortly after the application of treatments. Preranking and blocking, however, would be less advantageous the longer the time between treatment application and evaluation of the treatment.

Figure 4B shows the Spearman correlation coefficients for trap catch on a given date compared with catch on the prior observation date. For this short duration (3- to 8-d period), the correlation in trap numbers remained high over the entire season, generally ranging between *r* = 0.8 and *r* = 0.7. Only on 10 July 2020 and 30 July 2020 did the correlation drop to below *r* = 0.5.

Based on the correlations between the initial pretreatment count per trap and the quick drop-off in correlation between trap captures in untreated control plots as the season progressed, the use of pretreatment assessments appeared to work best when posttreatment assessments occurred within a few days after the treatment was applied.

### Comparison Between Study Sensitivity Using a Completely Random Design Versus Blocked Assignment of Treatments

**Fig. 5.**
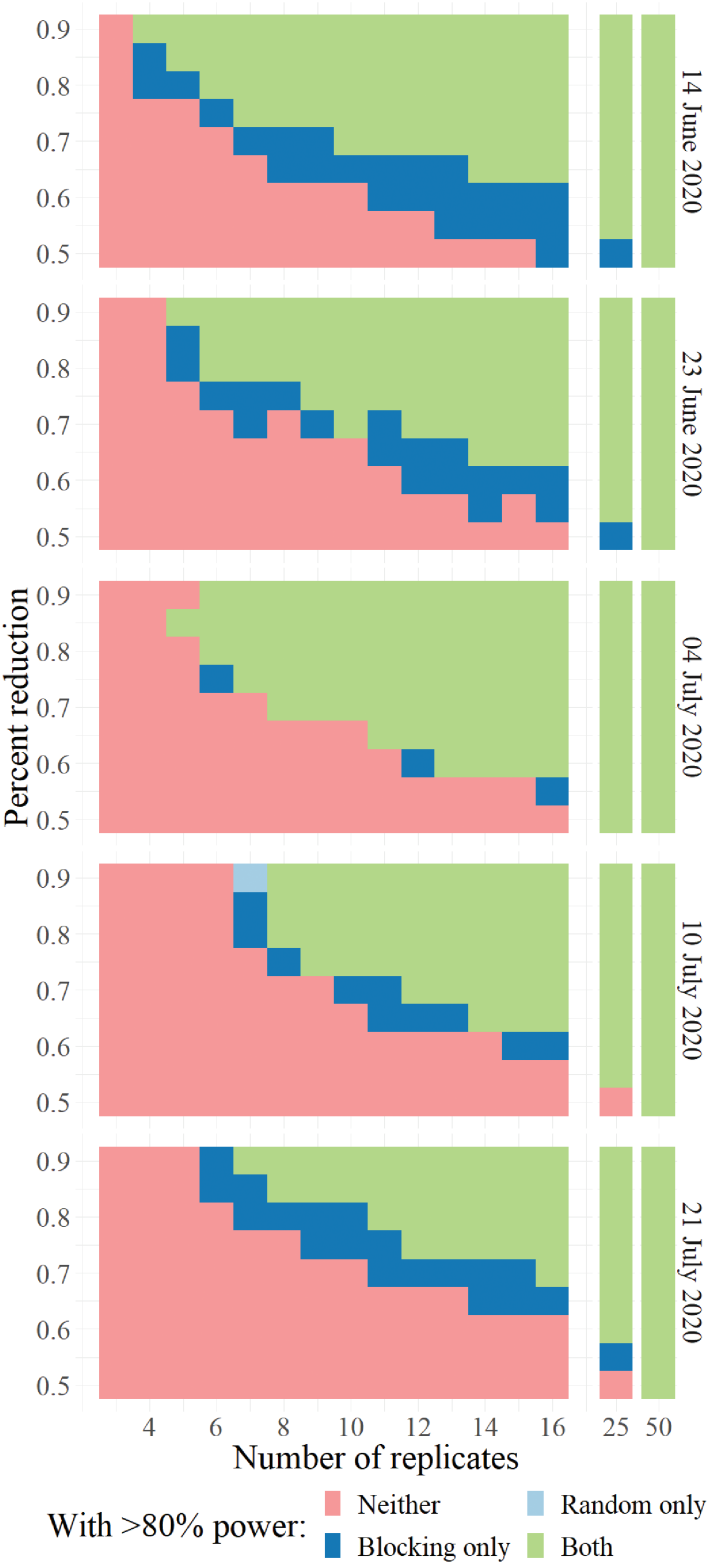
Experimental conditions under which 80% power was achieved by sampling spotted lanternfly using sticky bands and employing either a completely random experimental design or an experiment with blocks determined by pretreatment spotted lanternfly abundance. Each cell is color-coded to denote which experimental design(s) resulted in greater than 80% of simulation runs correctly assessing that there is a significant difference between treated and control plots. Green cells indicate both designs had >80% power, dark blue cells that the blocked design had >80% power while the random design did not, pale blue cells that the completely random design had power over 80% while the blocked design did not, and pink cells that neither design resulted in power over 80%.

**Fig. 6.**
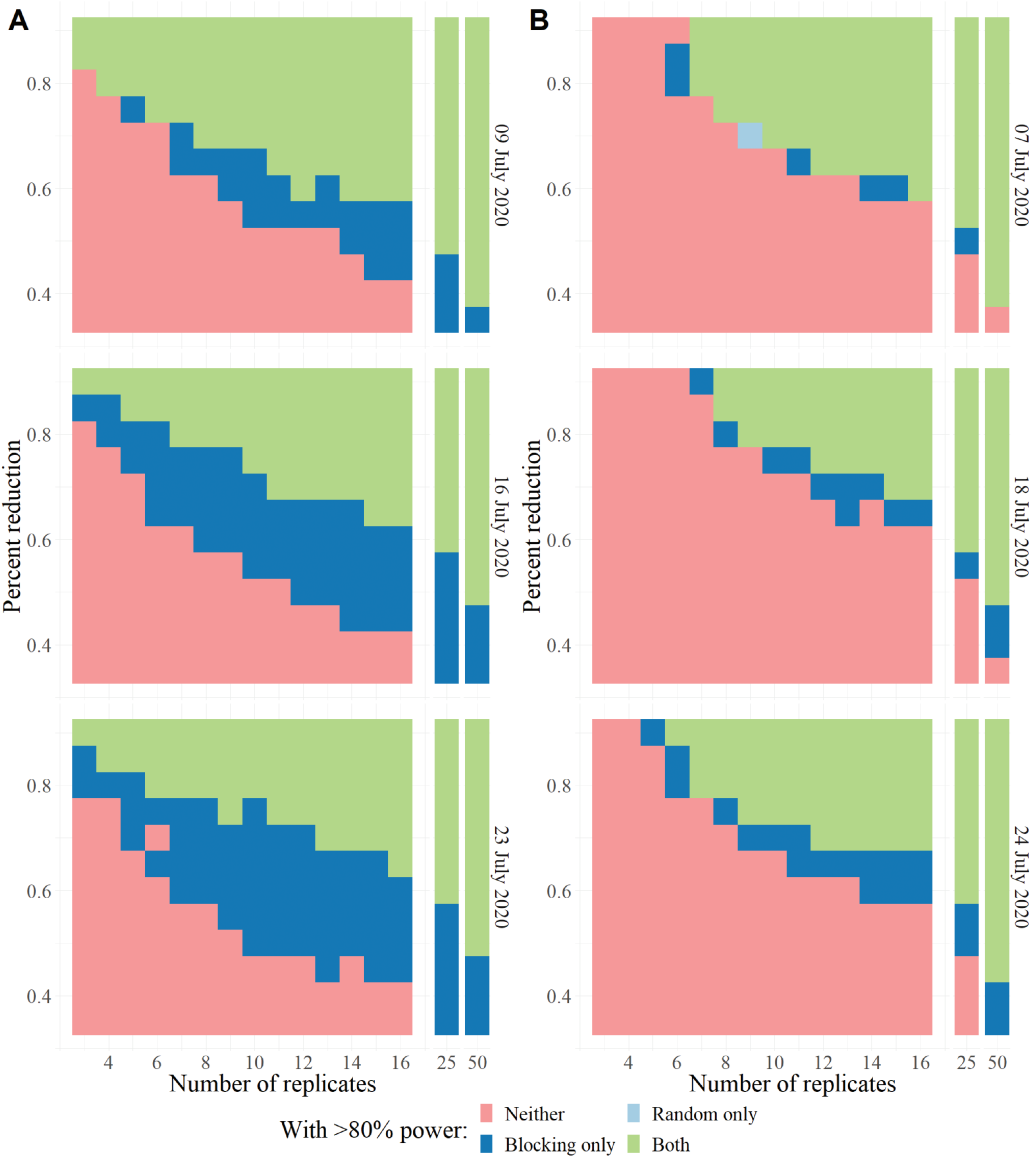
Experimental conditions under which 80% power was achieved by sampling using 20 min of in situ visual counts per plot (A) or using a single BugBarrier sticky band trap per plot (B), on comparable dates. Each cell is color-coded to denote which experimental design(s) resulted in greater than 80% of simulation runs correctly assessing that there is a significant difference between treated and control plots. Green cells indicate both designs had >80% power, dark blue cells that the blocked design had >80% power while the random design did not, pale blue cells that the completely random design had power over 80% while the blocked design did not, and pink cells that neither design resulted in power over 80%.

In general, blocking a study based on pretreatment mean density assessments and posttreatment assessments within a few days of treatment provided an improvement in study sensitivity (Figs. 5 and 6). Based on sticky band capture data, simulation of a pretreatment assessment on 11 June and posttreatment assessment on 14 June 2020 indicated that blocking the study would enable the experiment to reliably detect a 60% reduction in mean count with nine replicates, whereas the random design could only detect a reduction of 70% or greater. For a pretreatment assessment on 20 June 2020 and posttreatment assessment on 23 June 2020, blocking improved sensitivity from 70 to 60% with 11 replications. If the preassessment occurred on 1, 7, or 18 July 2020 followed by a posttreatment assessment on 4, 10, or 21 July 2020, the study was able to discern reductions of 60–70% with 13, 15, and 10 samples (replications), respectively. It appears that the later in the season the study is conducted, the harder it is to improve study sensitivity to detect differences smaller than 70%. This corresponds to nymphs being primarily in the first and second instars on earlier dates of the experiment, and second, third, or fourth instars during the later dates (Table 8). Pretreatment assessment and ranking of plots for assignment to blocks improved the study sensitivity over a completely random assignment of treatment to the plots.

We observed that the blocked design achieved even larger improvements in statistical power when in situ visual searches were used to quantify spotted lanternfly abundance rather than sticky band captures (Fig. 6). With 10 replicates and assessment on 23 June 2020, for example, blocking achieved 80% power with effect sizes 25% less than those the completely random design was reliably able to detect.

While increasing the number of samples should lead to increased study sensitivity, even increasing the sample size to 25 replications (with increased labor and cost) would only allow experimenters to reliably detect reductions in mean counts of 45% or greater should they use sticky bands to measure spotted lanternfly abundance and the completely random design. Blocking and using in situ visual searches, however, provided 80% power with proportional reductions in mean spotted lanternfly counts as small as 35%. Without new, more time efficient sampling methods or clever experimental designs, however, feasible field studies will be limited in their ability to measure small treatment effects.

### Comparison of Sampling Efficiency Between Sticky Trap and In Situ Evaluation Methods for Use In Spotted Lanternfly Surveillance Programs

The cost of monitoring 20 sticky traps (trees) in the Wyomissing, PA housing association to count the total number of nymphs and separate then into instars was estimated as $41.67, $50.00, and $66.70 per sample date for wages of $12.50, $15.00, and $20.00 per hour, respectively. The estimated time to monitor and change out traps was around 10 min per trap at Wyomissing. Therefore, the cost per tree was around $2.08, $2.50, and $3.33 per sample date (trap) for the three wage levels. Between 1 June 2020 and 31 August 2020 (period of nymph activity), the total number of nymphs on the 20 trees ranged from 37,273 to 1 at the Wyomissing site. While the time needed to monitor each sticky trap was less at lower densities, the total time required per tree remained close to 10 min over the study. This was because the time spent traveling between individual trees, and removing and replacing traps was constant, while the number of people monitoring the traps was reduced from three to two individuals when densities were lower. For the 24 and 25 sampling dates, the cost of the time spent counting nymphs and changing traps was about $1,200 to $1,668.

Applying the average cost per trap (tree) from the Wyomissing study to the Blue Marsh Lake pesticide research study, we estimated a total cost to evaluate the 90 sticky traps of between $187.00 and $299.00 per sample date. The actual per tree (trap) monitoring time at Blue Marsh Lake was around 5 min and 24 s (5.4 min) for two people to count nymphs on the trap and change the trap. Therefore, the total time spent monitoring two sticky traps (trees) in each plot at Blue Marsh Lake was around 10 min and 48 s (10.8 min). This was about 48% less time than at Wyomissing, PA. Using the Blue Marsh Lake time of 5.4 min the total cost to collect count data during 2020 over 14 sample dates was between $1,257 and $2,010. These cost estimates did not include the time spent traveling between plot locations, which was significant at Blue Marsh Lake due to the area covered and limited road access to the individual sites. The two sites provide a general range of estimated time and cost to monitor sticky traps in two types of studies.

Each plot with two sticky traps required about 10–20 min of labor to monitor, while for the in situ visual counts, the time per plot was 20 min of labor, with four people each taking a 5-min count. Thus, the total time to monitor a plot using sticky trap counts versus in situ counts was from ½ to equal in time, depending on whether the Wyomissing or Blue Marsh Lake measured times were used. Given that the cost of labor is the same for sticky trap and in situ counts, the comparative cost of using sticky traps is ½ to equal that of visual counts. However, the cost of sticky traps would also include purchasing trap materials, which in situ counts do not require.

## Discussion

Analysis of catch on sticky band traps showed that all spotted lanternfly instars tended to be aggregated in rural and urban/suburban landscapes. The degree of aggregation tended to be slightly higher in rural areas (on *A. altissima*) than in the urban/suburban setting (on *A. rubrum*). Therefore, higher numbers of sticky traps on *A. altissima* were required to estimate the population mean density in the rural area. The application of insecticide did not appear to alter the degree of nymph aggregation, despite reducing overall spotted lanternfly abundance. Most sites showed that nymph populations followed an aggregated dispersion pattern, and this was true in both the rural and suburban settings investigated here. Only at low-density populations did any sites show a random dispersion pattern. The degree of aggregation of early instars followed that of egg masses (Keller et al. 2020). Therefore, the use of egg mass counts or sticky trap captures of nymphs may be a good way to preassess where spotted lanternfly population densities occur for assigning plots to treatment blocks or for pairs using a paired comparison.

Comparing the in situ (visual) counts versus the sticky trap approach for estimating spotted lanternfly nymph numbers revealed that both approaches share a similar sample size and time requirement to complete. The advantages of in situ counts include that there is no cost associated with purchasing traps and installing them and removing them when they become saturated with nymphs. Using traps, one must also wait for some time period to sample the insects before treatment (we used 3 d), whereas with visual counts, insecticide treatments or other decisions can be made immediately. Further, visual counts do not remove insects from the experiment, which may influence the populations’ declines over time that we observed. One potential argument for using sticky traps is that when only first and second instars are present, in situ counts can be difficult. Early instar nymphs are very small and tend to move onto a wide range of hosts (Liu 2019). In this study, on the dates when in situ counts were taken, third and fourth instars were primarily present. They were likely easier to see and were more aggregated than the first instars.

Because spotted lanternfly populations declined over time, this decline must be accounted for when estimating the degree of treatment effect (difference between the untreated control and treatment) that can be measured in a study (Bartlett 1936). Correlation between traps counts over time showed that the ranking of traps based on number of spotted lanternfly caught was highly correlated for the first few days but then declined quickly as the season progressed. However, the correlation remained above 45% between consecutive sampling dates. Thus, it appears that the value of pretreatment assessment to establish blocks likely declines as the time from the pretreatment assessment and posttreatment assessment increases.

Simulations comparing a completely random design against a blocked design with blocking based on spotted lanternfly abundance shortly before the application of treatment showed that blocking can improve statistical power. When sticky bands were used to quantify spotted lanternfly abundance, the use of blocks tended to enable experiments to reliably detect differences in mean spotted lanternfly abundance 10–20% smaller than those detected by the completely random design over the five dates tested. Over the range of sample sizes we considered, though, neither design reliably detected differences smaller than a 50% difference between the untreated control and treatment when sampling with sticky bands. We estimated that the sample size must exceed 16 traps per treatment to measure a 50% reduction or less using a blocked design. Simulated experiments using in situ visual counts to measure spotted lanternfly abundance generally had slightly higher power than those using sticky bands. The improvement with blocking was also larger for experiments using in situ visual counts. Blocked experiments were able to detect proportional reductions in number 25% smaller than those that could be reliably detected by completely random experiments later in the season with larger numbers of replicates.

Sites’ composition (host habitat) for spotted lanternfly nymphs was not uniform, which is consistent with the great heterogeneity in plant community composition across the landscape that is typical of areas where spotted lanternfly are found. Therefore, determining how to best define the area of a site was difficult, and somewhat arbitrary. This is in contrast to other studies that focus on uniform host areas, such as cornfields, orchards, grain bins, etc. (Midgarden et al. 1993, Campbell et al. 2002). Typically, these types of studies are looking at an insect that is crop-specific or contained in a defined space. In most cases, fields and orchards are made up of a single host that tends to be planted in systematic rows or homogenous plantings. This is not the case for many of the habitats spotted lanternfly occupies because this fulgorid prefers areas with host plants like *A. altissima* and *A. rubrum* but feeds on a diverse range of host species across its life stages. Spotted lanternfly has a known host range of over 65–70 plant species in North America (Barringer et al. 2015, Dara et al. 2015, Lee et al. 2019, Urban 2020). The breadth of hosts that it can be found on and/or feed on within an area is influenced by the types of hosts available. Therefore, it would be nearly impossible and cost-prohibitive to design a study to quantify the true dispersion pattern of spotted lanternfly nymphs across all possible host species within a landscape area. This study focused on two of its hosts most preferred by nymphs as surrogates to measure its relative abundance and dispersion pattern within the defined sites.

In conclusion, spotted lanternfly nymphal dispersion patterns demand fairly large sample sizes to precisely measure densities, and experiments aiming to test management practices under field conditions must account for variation in spotted lanternfly abundance across the landscape and over time to efficiently measure treatment effects. Both sticky traps and in situ visual counts provided a similar sensitivity level, though in situ counts provided slightly higher experimental power. Using pretreatment counts to rank plots and then assign treatments within blocks based on the rank of plots provided improved power, allowing experiments to reliably measure treatment differences of 10–20% less than those reliably detected by a random design. In prior work, the use of visual pretreatment counts and a split plot design revealed a significant difference between the control and a *Beauveria bassiana* bioinsecticide treatment in 2019 (Clifton et al. 2020). Using a block design and in situ visual search or sticky traps would provide a reasonable approach for field studies, but the researcher should not expect to reliably measure treatment differences that are less than 40% given the spatial heterogeneity of spotted lanternfly nymphal dispersion.

## Supplementary Material

nvab104_suppl_Supplementary-Material

## References

[CIT0001] Barringer, L., and C. M. Ciafré. 2020. Worldwide feeding host plants of spotted lanternfly, with significant additions from North America. Environ. Entomol. 49: 999–1011. 32797186 10.1093/ee/nvaa093

[CIT0002] Barringer, L., L. R. Donovall, S. Scichiger, D. Lynch, and D. Henry. 2015. The first new world record of *Lycorma delicatula* (Insecta: Hemiptera: Fulgoridae). Entomol. News. 125: 20–23.

[CIT0003] Bartlett, M. S . 1936. Some notes on insecticide tests in the laboratory and in the field. Suppl. J. R. Stat. Soc. 3: 185–194.

[CIT0004] Brockerhoff, E. G., A. M. Liebhold, B. Richardson, and D. M. Suckling. 2010. Eradication of invasive forest insects: concepts, methods, costs and benefits. N. Z. J. For. Sci. 40(suppl.): S117–S135.

[CIT0005] Brooks, M. E., K. Kristensen, K. J. van Benthem, A. Magnusson, C. W. Berg, A. Nielsen, H. J. Skaug, M. Maechler, and B. M. Bolker. 2017. glmmTMB balances speed and flexibility among packages for zero-inflated generalized linear mixed modeling. R J. 9: 378–400.

[CIT0006] Brown, J. L., and G. H. Orians. 1970. Spacing patterns in mobile animals. Annu. Rev. Ecol. Syst. 1: 239–262.

[CIT0007] Bruck, D. J., M. Bolda, L. Tanigoshi, J. Klick, J. Kleiber, J. DeFrancesco, B. Gerdeman, and H. Spitler. 2011. Laboratory and field comparisons of insecticides to reduce infestation of *Drosophila suzukii* in berry crops. Pest Manag. Sci. 67: 1375–1385. 21800409 10.1002/ps.2242

[CIT0008] Calvin, D. D., M. C. Knapp, K. Xingquan, F. L. Poston, and S. M. Welch. 1986. Using a decision model to optimize European corn borer (Lepidoptera: Pyralidae) egg-mass sampling. Environ. Entomol. 15: 1212–1219.

[CIT0009] Campbell, J. F., M. A. Mullen, and A. K. Dowdy. 2002. Monitoring stored-product pests in food processing plants with pheromone trapping, contour mapping, and mark-recapture. J. Econ. Entomol. 95: 1089–1101. 12403439 10.1603/0022-0493-95.5.1089

[CIT0010] Champely, S . 2020. pwr: basic functions for power analysis. R package version 1.3-0. https://CRAN.R-project.org/package=pwr.

[CIT0011] Clifton, E. H., A. E. Hajek, N. E. Jenkins, R. T. Roush, J. P. Rost, and D. J. Biddinger. 2020. Applications of *Beauveria bassiana* (Hypocreales: Cordycipitaceae) to control populations of spotted lanternfly, *Lycorma delicatula* (Hemiptera: Fulgoridae), in semi-natural landscapes and on grapevines. Environ. Entomol. 49:854–864. 32488261 10.1093/ee/nvaa064

[CIT0012] Dara, S., L. Barringer, and S. Arthurs. 2015. *Lycorma delicatula* (Hemiptera: Fulgoridae): a new invasive pest in the United States. J. Integr. Pest Manag. 6: 20.

[CIT0013] Elliot, J. M . 1977. Some methods for the statistical analysis of samples of benthic invertebrates. Freshwater Biological Association, Ambleside, United Kingdom.

[CIT0014] Francese, J. A., M. F. Cooperband, K. M. Murman, S. L. Cannon, E. G. Booth, S. M. Devine, and M. S. Wallace. 2020. Developing traps for the spotted lanternfly, *Lycorma delicatula* (Hemiptera: Fulgoridae). Environ. Entomol. 49: 269–276. 31990325 10.1093/ee/nvz166

[CIT0500] Hardin, J. W., and J. M. Hilbe. 2007. Generalized linear models and extensions, 2nd ed. Stata Press, College Station, TX.

[CIT0015] Harper, J., W. Stone, T. Kelsey, and L. Kime. 2019. Potential economic impact of the spotted lanternfly on agriculture and forestry in Pennsylvania. Center for Rural Pennsylvania, Harrisburg, PA. https://www.rural.palegislature.us/documents/reports/Spotted-Lanternfly-2019.pdf.

[CIT0016] Iwao, S . 1968. A new regression method for analyzing the aggregation pattern of animal populations. Popul. Ecol. 10: 1–20.

[CIT0017] Iwao, S., and E. Kuno. 1968. Use of regression of mean crowding on mean density for estimating sample size and the transformation of data for the analysis of variance. Popul. Ecol. 10: 210–214.

[CIT0018] Keller, J., J. Rost, K. Hoover, J. Urban, H. Leach, M. Porras, B. Walsh, M. Bosold, and D. Calvin. 2020. Dispersion patterns and sample size estimates for egg masses of spotted lanternfly (Hemiptera: Fulgoridae). Environ. Entomol. 49: 1462–1472. 33315076 10.1093/ee/nvaa107

[CIT0019] Leach, H., and A. Leach. 2020. Seasonal phenology and activity of spotted lanternfly (*Lycorma delicatula*) in eastern US vineyards. J. Pest Sci. 93: 1215–1224.

[CIT0501] Leach, H., D. J. Biddinger, G. Krawczyk, E. Smyers, and J. M. Urban. 2019. Evaluation of insecticides for control of the spotted lanternfly, Lycorma delicatula, (Hemiptera: Fulgoridae), a new pest of fruit in the Northeastern US. Crop Prot. 124: 104833.

[CIT0020] Lee, D. H., Y. L. Park, and T. C. Leskey. 2019. A review of biology and management of *Lycorma delicatula* (Hemiptera: Fulgoridae), an emerging global invasive species. J. Asia Pac. Entomol. 22: 589–596.

[CIT0021] Liu, H . 2019. Oviposition substrate selection, egg mass characteristics, host preference, and life history of the spotted lanternfly (Hemiptera: Fulgoridae) in North America. Environ. Entomol. 48: 1452–1468. 31651025 10.1093/ee/nvz123

[CIT0022] Lloyd, M . 1967. Mean crowding. J. Anim. Ecol. 36: 1–30.

[CIT0023] Midgarden, D. G., R. R. Youngman, and S. J. Fleischer. 1993. Spatial analysis of counts of western com rootworm (Coleoptera: Chrysomelidae) adults on yellow sticky traps in corn: geostatistics and dispersion indices. Environ. Entomol. 22: 1124–1133.

[CIT0024] Morisita, M . 1959. Measuring of the dispersion of individuals and analysis of the distributional patterns. Mem. Fac. Sci. Kyushu Univ., Ser. E (Biol.) 2: 215–235.

[CIT0025] (NYSIPM) New York State Integrated Pest Management. 2021. Spotted lanternfly. https://nysipm.cornell.edu/environment/invasive-species-exotic-pests/spotted-lanternfly/. Accessed 5 May 2021.

[CIT0026] R Development Core Team. 2020. R: a language and environment for statistical computing, version 3.6.3. R Foundation for Statistical Computing, Vienna, Austria.

[CIT0027] Sabbatini Peverieri, G., F. Binazzi, L. Marianelli, and P. F. Roversi. 2018. Lethal and sublethal effects of long-lasting insecticide-treated nets on the invasive bug *Halyomorpha halys*. J. Appl. Entomol. 142: 141–148.

[CIT0028] Southwood, T. R. E . 1971. Ecological methods with particular reference to the study of insect populations. Chapman & Hall, London, United Kingdom.

[CIT0029] Urban, J. M . 2020. Perspective: shedding light on spotted lanternfly impacts in the USA. Pest Manag. Sci. 76: 10–17. 31525270 10.1002/ps.5619

[CIT0030] Waters, E. K., M. J. Furlong, K. K. Benke, J. R. Grove, and A. J. Hamilton. 2014. Iwao’s patchiness regression through the origin: biological importance and efficiency of sampling applications. Popul. Ecol. 56: 393–399.

