# Supplementary material for: Spotted Lanternfly (Hemiptera: Fulgoridae) Nymphal Dispersion Patterns and Their Influence on Field Experiments
Source: Environ Entomol. 2021 Sep 23;50(6):1490–504. doi: 10.1093/ee/nvab104 (PMC8678449; doi:10.1093/ee/nvab104)
Supplement: nvab104_suppl_Supplementary-Material [file nvab104_suppl_supplementary-material.docx]

**Supplemental Material**

**Spotted Lanternfly (Hemiptera: Fulgoridae) Nymph Dispersion Patterns and Their Influence on Field Experiments**

**Authors:** Calvin, D. D.^1^, J. Keller^2^, J. Rost^3^, B. Walsh^4^, D. Biddinger^5^, K. Hoover^2^, B. Treichler^6^ A. Johnson^2,^ and R. T. Roush^1^

^1^Office of the Dean, The Pennsylvania State University, University Park, PA 16802

^2^Department of Entomology, The Pennsylvania State University, University Park, PA 16802

^3^Tulpehocken Road, P.O. Box 7009, Penn State Berks Campus, Reading, PA 19610

^4^Penn State Extension Berks County Office, 1238 County Welfare Rd # 110, Leesport, PA 19533

^5^Fruit Research and Extension Center, P.O. Box 330, Biglerville, PA 17307-0330

^6^U.S. Army Corp of Engineers, Blue Marsh Lake, 1268 Palisades Drive, Leesport, PA 19533

*Simulation approach to test the effect of blocking on SLF insecticide experimental power.*

As described in the text, this is a 7-step process:

1. Characterize the distribution of SLF trap counts on the pre-treatment date in field collected data.
2. Simulate initial counts for the desired number of simulated plots.
3. Assign each simulated plot to either the control or to the treatment in two ways: completely random or by blocking based on initial counts.
4. Describe the transition from counts on the pre-treatment date to counts on the evaluation date based on field collected data.
5. Generate post-treatment counts for simulated plots under each experimental design.
6. Fit the negative binomial mixed effects regression model to the simulated data for the evaluation date and determine whether the model correctly identifies the difference between treated and control plots.
7. Repeat this process 300 times and determine the frequency with which a significant difference between the simulated treated plots and simulated control plots was detected under each experimental design.

Step 1: To begin, we characterized the distribution of counts in untreated control plots from the field study. Using the *glmmTMB* function from the *glmmTMB* library in R (Brooks et al 2017), we fit a generalized linear model with a negative binomial error structure (family=nbinom1 in the function) to these counts, fitting the model with only a y-intercept. This model gave the estimated mean and the dispersion parameter for the negative binomial distribution that best fit the data (Figure S1A).

Step 2: We then generated simulated initial SLF counts for the desired number of simulated plots using the *rnbinom* function in r, with the mean equal to that given in the regression from step one and size equal to that mean divided by the overdispersion parameter from the fitted model, as is appropriate for the linear parameterization of the negative binomial distribution (Figure S1B).


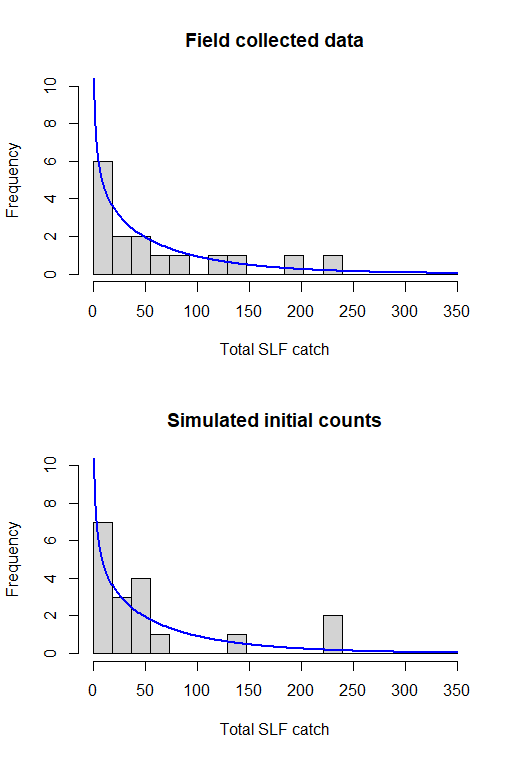


**Figure S1**. Histogram showing the distribution of SLF trap counts on 23 June, 2020 (A) with the fitted negative binomial distribution shown in blue, and a single iteration of simulating 18 plots based on this distribution (B).

Step 3: Each simulated plot was then assigned to either the treatment or the control under the completely random design by allocating half to be treated and half to be untreated control plots at random. Then, each simulated plot was assigned to the treatment or control under the blocked design by sorting all plots by their initial count, assigning each sequential pair of plots to a block together, and then randomly assigning 1 plot in each block to be the untreated control and the other to be treated.

Step 4: We described the transition from count on the pre-treatment date to count on the assessment date by fitting a generalized linear model with negative binomial error distribution to data from control plots in the field experiment. Details describing these regressions are contained in Table S1. We used the function *r.squaredLR* from the *MuMIn* package in R to estimate r-squared values for these regressions. This method is one proposed way to calculate pseudo-R^2^ values for nonlinear models such as those we fit here.

**Table S1**: Fitted regression parameters and standard errors for generalized linear models with negative binomial error structure regressing trap catch on the evaluation date against the same trap’s catch on the pre-treatment date in untreated control plots in the Blue Marsh Lake Recreation Area in summer 2020.

| **Pre-treatment date** | **Evaluation date** | **Days between** | **Intercept** | **Intercept Std. Error** | **Slope** | **Slope Std Error** | **Adj. R-squared value** |
| --- | --- | --- | --- | --- | --- | --- | --- |
| 11-Jun-20 | 14-Jun-20 | 3 | 2.92 | 0.24 | 0.00656 | 0.00098 | 0.6 |
| 14-Jun-20 | 20-Jun-20 | 6 | 3.2 | 0.25 | 0.0125 | 0.0024 | 0.53 |
| 20-Jun-20 | 23-Jun-20 | 3 | 3.16 | 0.29 | 0.0113 | 0.0018 | 0.59 |
| 23-Jun-20 | 26-Jun-20 | 3 | 2.75 | 0.29 | 0.011 | 0.0017 | 0.89 |
| 26-Jun-20 | 4-Jul-20 | 8 | 2.86 | 0.26 | 0.0064 | 0.0026 | 0.2 |
| 4-Jul-20 | 7-Jul-20 | 3 | 2.87 | 0.31 | 0.0037 | 0.0059 | 0.02 |
| 7-Jul-20 | 10-Jul-20 | 3 | 2.08 | 0.31 | 0.02 | 0.0058 | 0.34 |
| 10-Jul-20 | 18-Jul-20 | 8 | 2.21 | 0.34 | 0.039 | 0.01 | 0.38 |
| 18-Jul-20 | 21-Jul-20 | 3 | 2.56 | 0.3 | 0.034 | 0.0054 | 0.62 |
| 21-Jul-20 | 24-Jul-20 | 3 | 2.12 | 0.26 | 0.014 | 0.0027 | 0.51 |
| 24-Jul-20 | 27-Jul-20 | 3 | 1.1 | 0.28 | 0.034 | 0.0058 | 0.57 |
| 27-Jul-20 | 30-Jul-20 | 3 | 1.46 | 0.38 | 0.072 | 0.023 | 0.32 |


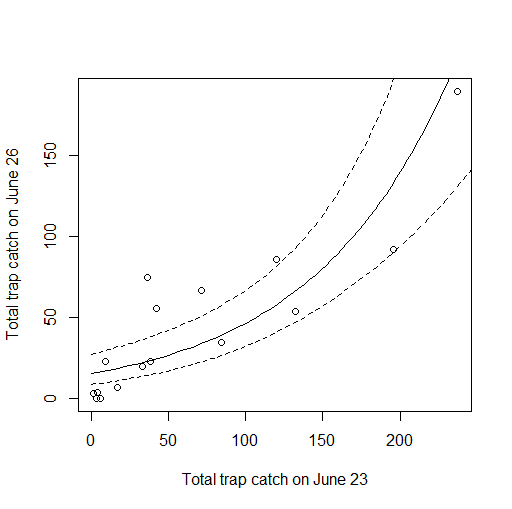


**Figure S2:** Trap catch on 26 June, 2020 plotted against trap catch on 23 June, 2020, with fitted regression line (solid) and 95% confidence intervals for the regression mean (dashed).

Step 5: In order to generate post-treatment counts for simulated control plots, we drew random values from the negative binomial distribution with mean equal to the fitted mean based on that plot’s initial count and size equal to that fitted mean divided by the overdispersion parameter from the regression. For treated plots, we multiplied the mean value by (1-*r*) where *r* is the percent reduction in mean for the hypothetical insecticide.


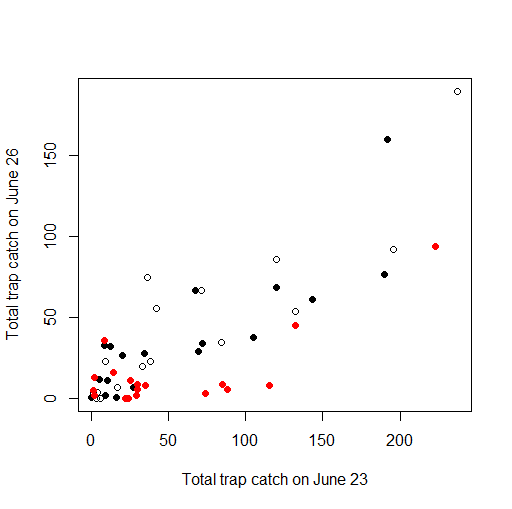


**Figure S3:** Trap catch on 26 June, 2020 plotted against trap catch on 23 June, 2020. Open points show actual field data, filled points show simulated untreated control data, and red points show simulated data for treated plots where the treatment reduced mean SLF catch by 60%. This is a single iteration of the simulation procedure.

Step 6: We assessed whether the simulated treated plots could be distinguished from the simulated untreated control plots by fitting a generalized linear model with negative binomial error structure predicting count on the evaluation date by treatment. Because we were interested *a priori* in investigating reductions in counts, we based the determination on a one-tailed z-test for the slope for treated plots testing whether this fitted parameter was significantly less than zero. The model for the blocked experimental design included a random intercept for each block.

Step 7: This process was repeated 300 times, and the number of times that the treatment was correctly found to have significantly lower counts than the control was recorded for each experimental design. The fraction of iterations where the correct assessment was made was the power for that experimental setup. We carried out simulations for a range of effect sizes and a range of levels of replication, carrying out 300 simulations for each unique combination of these two variables.

**Table S2**: Fitted regression parameters and standard errors for generalized linear models with negative binomial error structure regressing summed in situ visual search counts on the evaluation date against the same plot’s summed count on the pre-treatment date in untreated control plots in the Blue Marsh Lake Recreation Area in summer 2020. Note that in the regression count on the pre-treatment date was scaled by subtracting 650 to aid model convergence.

| **Pre-treatment date** | **Evaluation date** | **Days between** | **Intercept** | **Intercept Std. Error** | **Slope** | **Slope Std Error** | **Adj. R-squared value** |
| --- | --- | --- | --- | --- | --- | --- | --- |
| 2-Jul-20 | 9-Jul-20 | 7 | 6.47 | 0.18 | 0.00173 | 0.00048 | 0.57 |
| 9-Jul-20 | 16-Jul-20 | 7 | 6.82 | 0.15 | 0.00218 | 0.00044 | 0.74 |
| 16-Jul-20 | 23-Jul-20 | 7 | 6.15 | 0.18 | 0.00120 | 0.00018 | 0.78 |

**References:**

Brooks, M. E., K. Kristensen, K. J. van Benthem, A. Magnusson, C. W. Berg, A. Nielsen, H. J. Skaug, M. Maechler and B. M. Bolker. 2017. glmmTMB Balances Speed and Flexibility Among Packages for Zero-inflated Generalized Linear Mixed Modeling. The R Journal. 9(2): 378-400.
